# Thermotolerant *Campylobacter* spp. in chicken and bovine meat in Italy: Prevalence, level of contamination and molecular characterization of isolates

**DOI:** 10.1371/journal.pone.0225957

**Published:** 2019-12-06

**Authors:** Elisabetta Di Giannatale, Paolo Calistri, Guido Di Donato, Lucia Decastelli, Elisa Goffredo, Daniela Adriano, Maria Emanuela Mancini, Annamaria Galleggiante, Diana Neri, Salvatore Antoci, Cristina Marfoglia, Francesca Marotta, Roberta Nuvoloni, Giacomo Migliorati

**Affiliations:** 1 National Reference Laboratory for *Campylobacter*, Istituto Zooprofilattico Sperimentale dell'Abruzzo e del Molise "G. Caporale", Teramo, Italy; 2 National Reference Centre for Veterinary Epidemiology, Programming, Information and Risk Analysis (COVEPI), Istituto Zooprofilattico Sperimentale dell'Abruzzo e del Molise "G. Caporale", Teramo, Italy; 3 Dept. of Veterinary Sciences, Univ. of Pisa, Pisa, Italy; 4 Department of Food Hygiene, Istituto Zooprofilattico Sperimentale del Piemonte, Liguria e Valle d’Aosta, Torino, Italy; 5 Department of Food Hygiene, Istituto Zooprofilattico Sperimentale della Puglia e della Basilicata, Foggia, Italy; University of Campinas, BRAZIL

## Abstract

*Campylobacter* species are common foodborne pathogens associated with cases of human gastroenteritis worldwide. A detailed understanding of the prevalence, contamination levels and molecular characteristics of *Campylobacter spp*. in cattle and chicken, which are likely the most important sources of human contamination, is imperative. A collection of 1243 poultry meat samples (665 chicken breasts and 578 chicken thighs) and 1203 bovine meat samples (689 hamburgers and 514 knife-cut meat preparations) were collected at retail outlets, in randomly selected supermarkets located in different Italian regions during one year. Of these samples, 17.38% of the poultry meat and 0.58% of the bovine meat samples tested positive for *Campylobacter*, of which 131 were *Campylobacter jejuni* (57.96%) and 95 were *Campylobacter coli* (42.03%). *Campylobacter* isolates were genotyped with the aim of assessing the genetic diversity, population structure, source distribution and *Campylobacter* transmission route to humans. All isolates were molecularly characterized by pulse field gel electrophoresis (PFGE), and further genotyped using multilocus sequence typing (MLST) and fla-SVR sequencing to gain better insight into the population structure. Antibiotic resistance was also investigate. The highest levels of resistance among chicken strains were observed for ciprofloxacin (88.25%), nalidixic acid (81.45%) and tetracycline (75.6%). PFGE analysis revealed 73 pulsotypes for *C*. *jejuni* and 54 pulsotypes for *C*. *coli*, demonstrating the existance of different and specific clones circulating in Italy. MLST of *C*.*jejuni* isolates mainly clustered in the CC353, CC354, CC21, CC206 and CC443; while C.coli isolates clustered only in CC828. The most common flaA alleles were 287 for *C*. *jejuni* and 66 for *C*. *coli*. Our study confirms that poultry meat is the main source of *Campylobacter*iosis, whereas red meat had a low level of contamination suggesting a minor role in transmission. The high presence of *Campylobacter* in retail chicken meat, paired with its increased resistance to antimicrobials with several multidrug resistance profiles detected, is alarming and represents a persistent threat to public health.

## Introduction

*Campylobacter* is the most frequently reported zoonotic agent in the European Union (EU), with 246,158 cases reported in 2017 and an increasing trend of confirmed cases during the last year [1]. Official data published for Italy showed an apparent lower incidence of human infections compared with other EU countries. However, this difference is caused by a large underestimation of the true number of cases because the Italian reporting system on human infectious diseases does not collect etiological information on cases of gastroenteritis caused by a number of agents (*Campylobacter* included). The only data available on these infections are those reported voluntarily through Enter-Net, the international network for the surveillance of human gastrointestinal infections. While *Campylobacter* species rarely cause clinical disease in animals, they can produce severe acute gastroenteritis in humans [2]. *Campylobacter jejuni* and *Campylobacter coli* are the two predominant species causing gastrointestinal illness, although other species, such as *Campylobacter lari*, *Campylobacter upsaliensis* and *Campylobacter concisus*, have also been associated with gastrointestinal disorders in humans. Transmission of *Campylobacter* to humans occurs mainly through direct contact with live animals or by the consumption of contaminated foodstuffs, especially undercooked meat, unpasteurized milk, and untreated drinking water [3,4,5]. Poultry meat is considered the most important source of human *Campylobacter*iosis and the role of poultry as a reservoir for transmission to humans has been recognized. Around 20%–30% of human infections are linked to the manipulation, preparation and consumption of broiler meat, while 50%–80% may be attributed to the chicken reservoir as a whole [6]. In general, *Campylobacter* is less frequently associated with bovine meat in the EU. The proportion of *Campylobacter*-positive samples (single or batch) of fresh pig or fresh bovine meat was generally low [7]. In the EU, a harmonized and standardized baseline survey on the prevalence of *Campylobacter* in broiler flocks and broiler carcasses was carried out during 2008, allowing for estimation of the prevalence of contaminated animals by obtaining quantitative data on the level of *Campylobacter* contamination in broiler carcasses at slaughter. The results of this survey showed large variability among the EU Member States in the prevalence of *Campylobacter*-contaminated broiler carcasses, ranging from 5.5% to 100% (average of 75.8%, 95% CI = 73.2%–78.3%), with an estimated value of 49.6% (95% CI = 39.5%–59.7%) in Italy. No official data are currently available about the contamination of poultry and bovine meat in retail outlets in Italy. Several localized studies in Italy reported that the prevalence of thermophilic *Campylobacter* in fresh poultry meat and ready-to-cook products in retail outlets varied from 20% to 80% [8–13].

Campylobacter antimicrobial resistance has increased worldwide [14–16]. In particular, high levels of resistance to fluoroquinolones and macrolides in *C*. jejuni and C. coli isolates, as well as emerging resistance to aminoglycosides, have been reported both in human than in animal strains [17–19]. Although campylobacteriosis is generally a self-limiting disease and antibiotic treatment is not commonly required; sometimes, for patients with severe symptoms and/or compromised immunological systems, the antibiotic treatment is necessary. Fluoroquinolones, (especially ciprofloxacin) have long been considered the main treatment option [20]. However, due to high levels of resistance to this drug, macrolides are becoming the currently recommended first line of treatment for human. The subtyping of *Campylobacter* spp. isolates from different sources produces epidemiological linkage information that may be useful for identifying *Campylobacter* infection sources and controlling disease [21]. Generally, molecular typing is for differentiate between isolates of the same species of bacteria; while genotyping methods can be used to identify the genetic relatedness between different strains of bacteria. In order to track Campylobacter infections, various genotyping methods are used, such as pulsed-field gel electrophoresis (PFGE) and multilocus sequence typing (MLST). Among molecular methods, pulsed-field gel electrophoresis (PFGE) is a widely recognized standard technique, discriminatory, employed to subtype *Campylobacter*. However, it requires expensive equipment and complicated protocols and no standard methods for the interpretation and sharing of data with other scientists exist. Furthermore, the genetic variation among Campylobacter can becomes a problem when using PFGE for genotyping because of the existence of some strains not typable using either of the commonly used restriction enzymes SmaI or KpnI, which bring about questions as to the usefulness of PFGE with Campylobacter species [22,23]. Multilocus sequence typing (MLST) is an epidemiology tool for tracing the origin of *C*.*jejuni* strains from a weak clonal population [24]. MLST also has the discriminatory power to characterize hypervariable genomes, such as those of Campylobacter. Finally, the sequencing of the short variable region (SVR) of flaA (gene encoding flagellin A) is regarded as a cheap and easy genotyping method for discriminating among *Campylobacter* isolates [25]. Combining these molecular epidemiological methods allows for an in-depth analysis of the relationships among *Campylobacter* isolates. We conducted a one-year study, from June 2015 to June 2016, on chicken and bovine meat in retail in Italy. Aims of this study were to: (a) determine the prevalence and the contamination levels of *Campylobacter spp*. in poultry meat, and minced and knife-cut bovine meat preparations; (b) analyze the genetic relatedness of isolates by using molecular subtyping methods to identify the potential risks of *Campylobacter* spp. on humans and (c) investigate antimicrobial resistance to the most important antibiotics used for human illness.

## Materials and methods

### Sample selection and experimental design

A total of 1243 poultry meat samples (665 chicken breasts and 578 chicken thighs) and 1203 bovine meat samples (689 hamburgers and 514 knife-cut meat preparations) were collected at retail outlets, in randomly selected supermarket shops located in different Italian regions during the period from June 2015 to June 2016. The sampling program was designed based on three geographical macro-regions (three districts located in the northern, four in the central and two in the southern Italy).The samples were stratified according to the provinces and the number of inhabitants. The sample size was chosen to estimate the prevalence of contamination with a 95% confidence interval (CI) and an accuracy of ± 3%, considering an expected prevalence of 50%, and an additional 10% of samples were included to replace possible losses [26]. Samples were transported at 4°C to the laboratory and processed within 24 h. A value of expected prevalence equal to 50% was chosen because the available data on *Campylobacter* contamination in broiler meat is varying greatly with mean values close to 50%, and no reliable prevalence estimates are available for the contamination in bovine meat products.

### Microbiological analyses and antimicrobial susceptibility

The isolation and enumeration of *Campylobacter* spp. were performed according to EN ISO 10272 part 1 and part 2: 2006 methods [27, 28]. The isolates phenotypically identified as *Campylobacter* spp. were then confirmed at the species level by a multiplex PCR assay, as described by Wang and colleagues [29]. Strains used as positive controls were *Campylobacter coli* NCTC 11353, *Campylobacter fetus* ATCC 19438, *Campylobacter jejuni* ATCC 33291, Campylobacter *upsaliensis* NCTC 11541 and *Campylobacter lari* NCTC 11552. DNA was extracted using the Maxwell 16 tissue DNA purification kit (Promega Corporation, Madison, WI) according to the manufacturer’s instructions. A microbroth dilution method using Sensititre® custom susceptibility plates, EUCAMP 2 (Trek Diagnostic Systems, Biomedical Service, Venice, Italy), was applied to establish the minimum inhibitory concentrations (MICs). To evaluate the MICs, the Swin v3.2 software (TREK Diagnostic Systems) was used in accordance to European Committee on Antimicrobial Susceptibility Testing (EUCAST) guidelines epidemiological cut-off values (ECOFFs). Colonies were cultured on Columbia agar for 48 h in a microaerophilic atmosphere and were then inoculated into Mueller–Hinton broth supplemented with 5% lysed horse blood (TREK Diagnostic Systems Ltd. East Grinstead, United Kingdom) and dispensed into EUCAMP2 microtiter plates (Sensititre, reference EUCAMP2 TREK diagnostic Systems Ltd). The plates contained known scalar concentrations of the following antimicrobial substances: gentamicin (Gm) (0.12–16 μg/ml), streptomycin (S) (0.25–16 μg/ml), ciprofloxacin (Cip) (0.12–16 μg/ml), tetracycline (Te) (0.5–64 μg/ml), erythromycin (E) (1–128 μg/ml) and nalidixic acid (NA) (1–64 μg/ml) [30]. The plates were then incubated at 42°C in a microaerophilic atmosphere for 48 h. *C*. *jejuni* strain NCTC 11351 was included as a control for the MIC test.

## Molecular analyses

### Pulsed-field gel electrophoresis (PFGE)

PFGE was performed in accordance with the instructions of the 2013 U.S. PulseNet protocol for *Campylobacter* [31]. *C*. *jejuni* and *C*. *coli* strains were subcultured on Columbia agar at 41.5°C for 48 h in a microaerophilic atmosphere and were embedded in agarose blocks (Seakem Gold agarose, Lonza, Rockland, USA). The blocks were then lysed, washed and digested with 25 U of SmaI enzyme (Promega, Italy) at 25°C for 4 h. Salmonella serovar Branderup H9812 was used as a molecular weight standard. PFGE was performed using a Chef Mapper XA (BioRad Inc,Hercules,California,USA) with the following parameters: initial switch time of 6.75 s, final switch time of 35.38 s for 18 h at 6 V and 14°C in 0.5× TBE buffer (Sigma). After electrophoresis, the gel was stained with Sybr Safe DNA gel stain (Invitrogen, USA) and photographed using a transilluminator (Alpha Innotech, USA). Bionumerics v. 7.5 software (Applied Maths, Belgium) was used to analyze the PFGE fingerprinting profiles. The levels of similarity were calculated by the Dice correlation coefficient (tolerance position set at 1%) and the unweighted pair group mathematical average UPGMA clustering algorithm was used for cluster analysis of the PFGE pattern. PFGE clusters were defined at 95% similarity between macro restriction patterns. Untypable isolates were not included in the analysis.

### Multilocus sequence typing (MLST)

MLST was performed as reported by Dingle et al. (2001) for all *Campylobacter* isolates. MLST involved amplifying a segment of seven housekeeping genes: aspA (aspartase, 477 bp), glnA (glutamine synthase, 477 bp), gltA (citrate synthase, 402 bp), glyA (serine hydroxyl methyl transferase, 507 bp), pgm (phosphor glucomutase, 498 bp), tkt (transketolase, 459 bp) and uncA (ATP synthase, alpha subunit, 489 bp) to yield a total composite sequence length (for all seven loci) of 3,309 bp. PCRs and sequencing reactions were carried out in accordance with the guidelines on the *Campylobacter* MLST website (http://pubmlst.org/*Campylobacter*/). Briefly, purified PCR products were sequenced using an ABI PRISM BigDye® Terminator 3.1 Cycle Sequencing Kit (Applied Biosystems, Foster City, CA, USA) in accordance with the manufacturer’s instructions, and then they were analyzed by an ABI PRISM 3500 Genetic Analyzer (Applied Biosystems). The alleles, sequence types (STs) and clonal complexes (CCs) were identified using the MLST database available at http://pubmlst.org/*Campylobacter*. Novel alleles were submitted to the PubMLST C. jejuni/C. coli database curators for number assignment.

### FlaA-SVR

Typing was performed by amplifying the short variable region of the flaA gene (flaA-SVR) sequence using previously described primers [32,33], followed by sequencing of the PCR product. Amplification products were verified by gel electrophoresis. PCR products were purified using ExoSAP-IT reagent (GE Healthcare,Piscataway,N.J.,USA) and sequenced using BigDye Terminator v.3.1 cycle sequencing kit (Applied Biosystems, Darmstadt, Germany) in accordance with the manufacturer's instructions. After sequencing, DNA was further purified by ethanol precipitation using the kit Agencourt CleanSEQ® (Beckman Coulter, Brea, CA, USA). Sequencing products were analyzed on a Genetic Analyzer 3500® (Life Technologies, Paisley, UK). The nucleotide sequences were compared with the *Campylobacter* flaA database (http://pubmlst.org/*Campylobacter*/flaA) and allele numbers were assigned accordingly. Confirmed sequences were aligned using MEGA 4 software [34]. For strains with possible new flaA-SVR alleles, DNA trace files were submitted to the database administrator for confirmation.

### Statistical analysis

All of the data obtained from qualitative analyses were submitted for frequency distribution analysis (Fisher’s exact test and χ-square test) considering the sample categories. Significance was defined at the P<0.05 level. The observed distribution of *Campylobacter* spp. contamination levels (as logarithmic values of contamination and considering a value of 5 CFU/g for samples that were positive using the qualitative method but negative using the enumeration method, <10 CFU/g) were fitted to theoretical distributions by the maximum likelihood estimation method, using the R statistical software [35] with the fitdistrplus package. Differences in logarithmic values of *Campylobacter* spp. contamination between the chicken thigh and breast samples were tested by the Mann–Whitney U test. The genetic diversity and the comparison between the molecular methods used in this study were determined using the Simpson's diversity index and the adjusted Rand index [36].

## Results

### Prevalence and contamination levels

**Fig 1:**
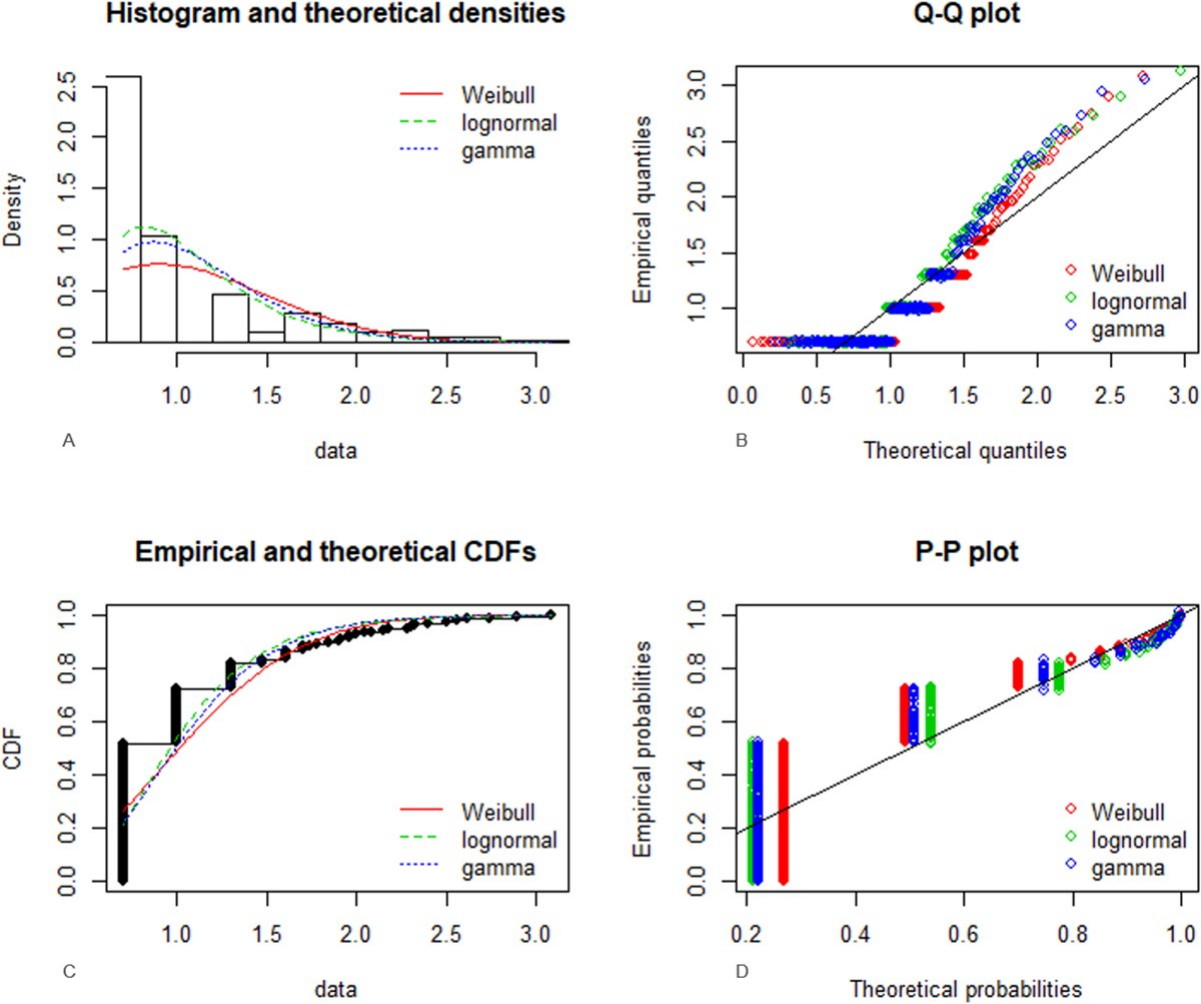
Results of the fitting of three main theoretical distributions: Weibull, lognormal and gamma on the logarithmic values of the Campylobacter spp. contamination levels in poultry meat samples. **A)** Histogram and theoretical densities (x: Log10); (y: Density). **B)** Q-Q plot compares the quantiles of Campylobacter contamination levels of distribution with the quantiles of three standardized theoretical distributions (Weibull, lognormal and gamma). **C)** Empirical and theoretical CDFs (x: Log10); (y: CDFs). **D)** P-P plot compares the empirical cumulative distribution function of Campylobacter contamination levels with Weibull, lognormal and gamma theoretical cumulative distribution function.

*Campylobacter* spp. were detected in 219 out of 1,243 (17.38%, 95% CI = 15.37%–19.58%) chicken meat samples and in 7 out of 1,203 (0.58%, 95% CI = 0.29%–1.19%) bovine meat samples (**Table 1**), with the prevalence in contaminated samples clearly higher in chicken meat (statistically significant difference by Fisher’s exact test, P<0.0001). The prevalence of *Campylobacter* was significantly (χ-square = 14.714, P<0.001) higher in chicken thighs (21.80%, 95% CI = 18.63%–25.35%) than in chicken breasts (13.53%, 95% CI = 11.15%–16.35%) **Table 1**. Out of 216 *Campylobacter*-positive poultry meat samples, 104 were confirmed to be positive by the enumeration method, with a quantification limit of 10 CFU/g, whereas only one sample of bovine hamburger was positive by the enumeration method at a level of 240 CFU/g. The distribution of the logarithmic values for *Campylobacter* spp. contamination levels in poultry meat samples was observed to fit three main distributions: Weibull, lognormal and gamma. The results of the fitting analysis are shown in **Fig 1** and the parameters of the fitted distributions are reported in **Table 2**. No differences in the levels of *Campylobacter* spp. contamination were observed between thigh (logarithm of the mean value: 1.47) and breast (logarithm of the mean value: 1.38) samples (Mann–Whitney U test, P = 0.485).

**Table 1:** *Campylobacter* spp. prevalence in poultry meat and bovine meat preparations.

Chicken samples	Tested samples	Positive samples	Prevalence (%)	95% C.I. (%)
Chicken thighs	578	126	21.80	18.63–25.35
Chicken breast	665	90	13.53	11.15–16.35
All poultry meat samples	1243	216	17.38	15.37–19.58
**Bovine samples**				
Hamburger	689	4	0.58	0.24–1.48
Italian traditional knife-cut meat preparation *	514	3	0.58	0.21–1.69
Total	1203	7	0.58	0.29–1.19

*carpaccio, Albese Knife-cut raw meat, salsiccia di Bra.

**Table 2:** Parameters for the fitted distribution on the logarithmic values of the *Campylobacter* spp. contamination levels in poultry meat samples.

	Estimated parameters		Loglikelihood	Akaike information criterion (AIC)	Bayesian information criterion (BIC)
	Distribution Parameters	Standard error			
Weibull	Shape = 2.185309 Scale = 1.193009	0.10398066 0.03957789	-140.8012	285.6024	292.353
Lognormal	Mean = -0.04043978 SD = 0.39914826	0.02715860 0.01920349	-99.3765	202.753	209.5036
Gamma	Shape = 5.699848 Rate = 5.422569	0.5331900 0.5302715	-116.097	236.1941	242.9446

### Species identification and antimicrobial resistance phenotypes

In total, 226 *Campylobacter* isolates were obtained: 128 *C*. *jejuni* (58.45%) and 91 *C*. *coli* (41.55%) isolates from chicken meat and three *C*. *jejuni* (42.85%) and four *C*. *coli* (57.14%) isolates in bovine meat. In 15 chicken meat samples (11 chicken thighs and 4 chicken breast), both *C*. *jejuni* and *C*. *coli* were isolated. Antimicrobial resistances are shown in **Table 3**. Around 88.25% of the *C*. *jejuni* and *C*. *coli* isolates were resistant to ciprofloxacin, 81.45% of the *C*. *jejuni* and *C*. *coli* isolates were resistant to nalidixic acid and 75.6% of *C*. *jejuni* and *C*. *coli* isolates were resistant to tetracycline. All tested *Campylobacter* isolates demonstrated low resistance to gentamicin and streptomycin. Moreover, we found a significantly higher percentage of resistance to erythromycin among *C*. *coli* (31.86%) isolates than among *C*. *jejuni* (14.06%) isolates (𝑃≤0.01, *𝜒*-square test). Multidrug resistance, defined as resistance to three or more unrelated antimicrobials, was respectively detected in 46.8% and 19.35% of *Campylobacter* isolates, resistant to ciprofloxacin, nalidixic acid and tetracycline (CipNaTe) and to ciprofloxacin, erythromycin, nalidixic acid and tetracycline (CipENATe).

**Table 3:** Breakpoint table for interpretation of MICs and percentage of *Campylobacter* isolates resistant to antimicrobials.

Antimicrobials	species	MIC breackpoint (μg/mL)		Percentage of *C*. *jejuni /C*. *coli* resistant (%)	Percentage of. *Campylobacter* spp. resistant (%)
		S	R		
Ciprofloxacin	*C*.*jejuni*	≤0.5	>0.5	87.5%	88,25%
	*C*.*coli*	≤0.5	>0.5	89.01%	
Erythromycin	*C*.*jejuni*	≤4	>4	14.06%	22,96%
	*C*.*coli*	≤8	>8	31,86%*	
Gentamicin	*C*.*jejuni*	≤2	>2	3.12%	3.76%
	*C*.*coli*	≤2	>2	4.4%	
Nalidixic acid	*C*.*jejuni*	≤16	>16	75%	81.45%
	*C*.*coli*	≤16	>16	87,91%	
Streptomycin	*C*.*jejuni*	≤2	>4	3.9%	7.45%
	*C*.*coli*	≤2	>4	10.99%	
Tetracycline	*C*.*jejuni*	≤2	>2	68.75%	75.6%
	*C*.*coli*	≤2	>2	82.42%	

S = sensible; R = resistant.

*statistically significant versus C. jejuni group (𝑃 ≤ 0.01, 𝜒2 test).

### PFGE, MLST and FlaA-SVR

PFGE analysis with SmaI of 124 strains of *C*. *jejuni* and 84 strains of C. coli showed 73 pulsotypes of *C*. *jejuni* and 54 pulsotypes of *C*. *coli*. A total of 5.6% of C.jejuni strains and 11.6% of C.coli resulted untypable. Using 95% similarity values, seven clusters for *C*. *jejuni* (A-B-C-D-E-F-G) and four clusters for *C*. *coli* (a,b,c,d) were obtained (S1 Fig; S2 Fig).

MLST typing of *C*. *jejuni* isolates detected 31 different STs belonging to 15 CCs and 11 STs without a defined CC (S3 Fig**)**. The four most frequent CCs detected were CC21, CC354, CC206 and CC353. Two STs, belonging to two of the five most common CCs, 2116ST (CC353) and 2863ST (CC354), were most frequently isolated from chicken breast samples. The only ST detected from a bovine hamburger isolate was 9140. MLST typing of *C*. *coli* indicated that these isolates were less diverse than *C*. *jejuni* isolates, with 81.4% of the meat isolates belonging to ST 828CC. The most numerous STs were 832 (CC828) from chicken meat and 7159 (CC828) from chicken meat and bovine hamburgers. In S4 Fig, the distribution frequency of *C*. *coli* CCs is reported. *C*. *coli* was also isolated from bovine hamburgers belonging to ST 3777 (CC828) and ST 2918, while one strain of *C*. *coli* isolated from a sausage belonged to S T4957 (CC828). Seventeen new STs were found in this study. The flaA typing revealed numerous alleles. The predominant flaA types were 287, 66 (in isolates from chicken meat and bovine hamburgers) and 34 (in isolates from chicken meat) (S5 Fig; S6 Fig). Through the analysis of PFGE, MLST and flagellin, the most prevalent circulating clone of *C*. *jejuni* was 2116ST-353complex with flaA 287, grouped in the major clonal population derived from PFGE (cluster C) formed by 24 isolates from poultry. The other two larger cluster of *C*.*jejuni* were formed (D and F), respectively, by six strains with genotype 2863ST-354 complex (flaA 34) and six strains with genotype 1039ST (flaA 551 and not available). The remaining clusters (A- B-E-G) included isolates with the genotypes: 400ST-353complex (flaA67); 2850ST-446complex (flaA 16) and 2833St-354complex (flaA34); 2863ST-354 complex (flaA 34) and 3335ST-206complex (flaA96) (S1 Fig).

The most prevalent circulating clones of *C*. *coli* were 832ST -828complex with flaA 17 and 7159 ST-828 complex with flaA 66, both formed by seven strains derived from PFGE and belonging to cluster b and d. The other clusters (a and c) were formed by STs 860 (flaA 30) and ST 827 (flaA 556) (S2 Fig).

## Discussion

*Campylobacter* contamination of broiler flocks on farms can lead to the transmission of these bacteria along the poultry production chain and result in poultry meat contamination at retail outlets. *Campylobacter* prevalence in poultry, as well as the contamination level of poultry products, varies greatly between different countries, justifying differences in the intervention strategies needed. The reduction of *Campylobacter*-positive chicken flocks, and consequently the prevalence and contamination levels of the meat, is the most effective strategy to reduce the number of human *Campylobacter* infections [37]. In our survey, 1,243 samples of chicken meat and 1,203 bovine meat samples were analyzed for *Campylobacter* spp. The prevalence of *Campylobacter*-contaminated samples among poultry meat was 17.38% (95% CI = 15.37%–19.58%), which was much higher than that observed among bovine meat samples (0.58%, 95% CI = 0.29%–1.19%). This confirms the substantial role of poultry-based meat products in human exposure to this pathogen. *C*. *jejuni* was the most common species identified in poultry meat. The limit of enumeration for *Campylobacter* was 10 CFU/g and only 104 poultry meat samples out of 216 samples (48.15%) positive for isolation were positive for enumeration, with 112 samples (51.85%) contaminated with less than 10 CFU/g. This confirms that in the majority of cases, the level of contamination of meat is low, as already observed in other studies [38–40]. Generally, poultry without skin, such as breast fillets, contain lower *Campylobacter* counts than portions with skin [41]. Significant differences can be detected among different typologies of poultry meat at retail outlets, in particular when the skin is removed, higher contamination was observed in thighs compared with whole breast or sliced meat. In our survey, *Campylobacter* was detected more frequently in portions with skin (thighs, 21.80%), than portions without skin (breast, 13.53%). These data agreed with the findings of other surveys. Bovines are also common carriers of *Campylobacter* but bovine meat is not considered the predominant vehicle of transmission in human infections, because *Campylobacter* is not commonly detected on the carcasses or in the meat at retail. In surveys of retail outlets worldwide, usually only 0%–10% of the samples test positive for *Campylobacter* [42–49]. In our study, the prevalence and levels of contamination of *Campylobacter* spp. in bovine meat were low, with a prevalence of 0.58% and only one sample contaminated with >10 CFU/g (240 CFU/g). *C*. *jejuni* and *C*. *coli* are becoming increasingly resistant to clinically important antibiotics, posing a major public health concern. High resistance rates to quinolones, fluoroquinolone and tetracycline among *Campylobacter* species have been reported in many countries but resistance to erythromycin and gentamicin in *C*. *jejuni* remains low [50,51]. Fluoroquinolones and macrolides (such as ciprofloxacin and erythromycin) are among the preferred antimicrobials for the treatment of human *Campylobacter* infections. Recent studies have shown a clear positive association between the use of fluoroquinolones in poultry production and increased resistance between chicken and human *Campylobacter* isolates [52]. In countries where the use of fluoroquinolones is not permitted in poultry production, such as Australia, Denmark, Finland and Sweden, few resistant *Campylobacter* strains have been isolated from chickens or humans [53,54]. Although macrolide resistance is relatively low among *C*. *jejuni* isolates, in recent years the increased prevalence of macrolide-resistant *Campylobacter* has been detected in certain regions of the world, such as the United States and Europe, and this trend is especially clear among *C*. *coli* isolates [55,56]. In our study, the highest level of resistance was observed against ciprofloxacin and nalidixic acid in most *Campylobacter* strains, and *C*. *coli* isolates were more frequently resistant to erythromycin than *C*. *jejuni* isolates. Multidrug resistance, defined as resistance to at least three different antimicrobials, was observed in 46.8% and 19.35% of the *Campylobacter* spp. strains (CipAnTe and CipAnTeE, respectively). PFGE, MLST and fla-typing are methods commonly used to study the distribution of different genotypes in various reservoirs and sources [57–58]. PFGE is considered the “gold standard” in molecular typing for most bacteria, as the entire genome is analyzed [59–60], but limitations of PFGE for the routine surveillance of *C*. *jejuni* have been reported [61]. Furthermore, this method requires expensive equipment, complicated protocols, and there are no standard methods for data interpretation or sharing these data with other scientists [62]. In our study, PFGE revealed significant differences in the Italian *C*. *jejuni* population. PFGE analysis with SmaI of 124 strains of *C*. *jejuni* and 84 strains of *C*. *coli* revealed high heterogeneity (73 pulsotypes for C. jejuni and 54 pulsotypes for *C*. *coli*). Using 95% similarity values, seven clusters were detected for *C*. *jejuni* and four clusters were detected for *C*. *coli*. Three of the seven *C*. *jejuni* clusters were the most numerous, consisting of 6, 6 and 24 strains characterized by the same MLST allelic profile and flaA allele ranging from 83% to 100%. The genotypes belonging to the individual clusters were 1039ST, 2863ST-354 complex and 2116ST-353 complex, respectively. All isolates of the three clusters were collected from central and southern regions of Italy. Among the *C*. *coli* isolates, three clusters composed of 7, 4 and 7 strains, were characterized with the same genotype, 832ST-828 complex, 827ST-828complex and 7159ST- 828 complex, respectively. Two clusters were composed of strains isolated in the center of Italy, while the third was the only cluster with strains isolated in the center and in the north of Italy. Molecular typing, by MLST, proved to be a helpful tool for investigating the relatedness of *Campylobacter* isolates from different sources [63]. It is highly reproducible, can easily be compared between different studies and a public database is available at PubMLST (http://pubmlst.org/*Campylobacter*/). Another genotyping method used less frequently for *Campylobacter* is fla-typing, which is based on the sequence of the SVR of the flagellin-encoding gene flaA (or flaB) and has a discriminatory index comparable to MLST [64,65]. Some *C*. *jejuni* CCs are strongly associated with an animal species or geographic area (specialist lineage), while others can colonize a wide range of host animals and reservoirs worldwide (generalist lineage). For example, ST257 and ST61 CCs are strongly associated with chickens and ruminants, respectively, while ST21 CC appeared to be characterized by phenotypic flexibility and high genetic microdiversity, revealing the properties of a generalist lineage, such as ST45 and ST828 [66,67]. In the present study, MLST analysis revealed that the most prevalent CCs for *C*. *jejuni* in poultry samples were 353 and 354, which was in accordance with the results obtained from studies in other countries [68–70], followed by CC21, 45, 206 and 443 with 20 different STs. The most prevalent MLST profile for *C*. *coli* was 832ST-828 complex. At the ST level, ST2116 (CC353) and ST2863 (CC354) were the most prevalent; they are clones circulating throughout the Italian territory that have been detected in all of the sampled regions. ST2116 was identified as a ST characteristic of Italian poultry that is not isolated in other European countries. The flaA gene of *Campylobacter* spp. serves as an epidemiological marker, as it shows extensive sequence heterogeneity. Seventy-one flaA-SVR types were identified among the numerous isolates that were analyzed, demonstrating the presence of a heterogeneous population. This was consistent with previous studies in which isolates from different continents revealed a similar degree of diversity [71].

## Conclusions

Our study confirms that poultry meat is the main source of *Campylobacter*iosis in humans, while bovine meat appears to play a minor role in the transmission of the disease. Our findings underline the importance of increasing farm biosecurity to reduce the level of contamination, to which consumers may be exposed, and to educate consumers to limit the risk of infection. Traditional molecular typing methods (PFGE, MLST, flaA) proved to be helpful tools in epidemiological investigations. Further studies should be conducted with the aim of establishing exact epidemiological links between the strains isolated from poultry meat chains and human isolates. Furthermore, MLST typing may be useful for comparing our isolates with those circulating in other countries. Overall, poultry is an important reservoir and source of human *Campylobacter*iosis, although the contribution of other sources, reservoirs and transmission routes warrants further research. Particularly worrying is the increase in the frequency of resistance against fluoroquinolones, quinolone and tetracycline. The systematic monitoring of antimicrobial resistance in *Campylobacter* spp. and of antimicrobial usage would be valuable in developing suitable strategies to control antimicrobial misuse and in monitoring the effectiveness of such strategies.

## Supporting information

S1 Fig Dendrogram of C. coli isolates in Italy (2015–2016). PFGE cluster analysis of a Sma I restriction enzyme digest of genomic DNA from C. coli isolates analyzed at a 95% similarity cut off. In the image, only the mayor clusters (a, b, c, and d) are shown.
(TIF)

S2 Fig Dendrogram of C. jejuni isolates in Italy (2015–2016). PFGE cluster analysis of a Sma I restriction enzyme digest of genomic DNA from C. jejuni isolates analyzed at a 95% similarity cut off. In the image, only the mayor clusters (A, B, C, D, E, F, and G) are shown.
(TIF)

S3 Fig Frequency and Italian distribution of *C.jejuni* clonal complex Sequence types (STs) among *C.jejuni* isolates from food animals (see the legend) are ordered by clonal complex (CC) and frequencies are represented by histograms. The major clonal complexes are indicated in the figure. The capital letters above the histograms indicate the Italian regions of origin (N = Northern Italy, which includes Piemonte and Liguria regions; C = Central Italy, which includes Abruzzo, Lazio and Marche regions; S = Southern Italy, which includes Puglia and Basilicata regions). NA = not assigned. New STS found in this study are indicated in asterisk.
(TIF)

S4 Fig Frequency and Italian distribution of *C.coli* clonal complex Sequence types (STs) among *C. coli* isolates from food animals (see the legend) are ordered by clonal complex (CC) and frequencies are represented by histograms. The capital letters above the histograms indicate the Italian regions of origin (N = Northern Italy, which includes Piemonte and Liguria regions; C = Central Italy, which includes Abruzzo, Lazio and Marche regions; S = Southern Italy, which includes Puglia and Basilicata regions). NA = CC not yet assigned. New STS found in this study are indicated in asterisk.
(TIF)

S5 Fig Distribution of FlaA alleles grouped by clonal complex corresponds to *Campylobacter coli*. (TIF)

S6 Fig Distribution of FlaA alleles grouped by clonal complex corresponds to *Campylobacter jejuni*. (TIF)

S1 File Dataset. Prevalence_Calculation: Campylobacter spp. prevalence in poultry meat and bovine meat preparations. Stat_analyses: Parameters for the fitted distribution on the logarithmic values of the Campylobacter spp. contamination levels in poultry meat samples. MIC_Calculation: Breakpoint table for interpretation of MICs and percentage of Campylobacter isolates resistant to antimicrobials.
(XLSX)
