# Supplementary figures and images for: Thermotolerant Campylobacter spp. in chicken and bovine meat in Italy: Prevalence, level of contamination and molecular characterization of isolates
Source: PLoS One. 2019 Dec 6;14(12):e0225957. doi: 10.1371/journal.pone.0225957 (PMC6897410; doi:10.1371/journal.pone.0225957)

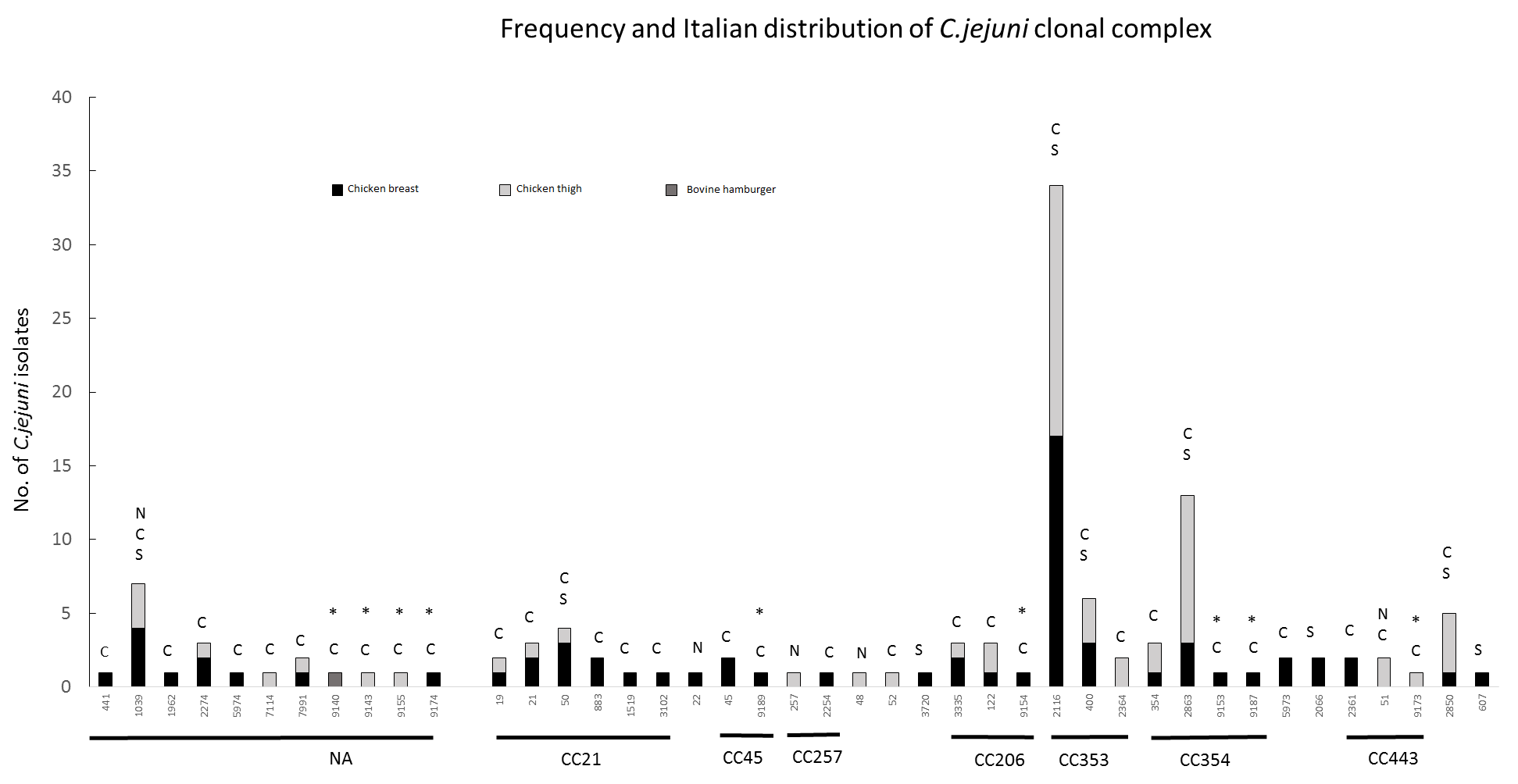

Supplement: S3 Fig — The major clonal complexes are indicated in the figure. The capital letters above the histograms indicate the Italian regions of origin (N = Northern Italy, which includes Piemonte and Liguria regions; C = Central Italy, which includes Abruzzo, Lazio and Marche regions; S = Southern Italy, which includes Puglia and Basilicata regions). NA = not assigned. New STS found in this study are indicated in asterisk. (TIF) [file pone.0225957.s003.tif]

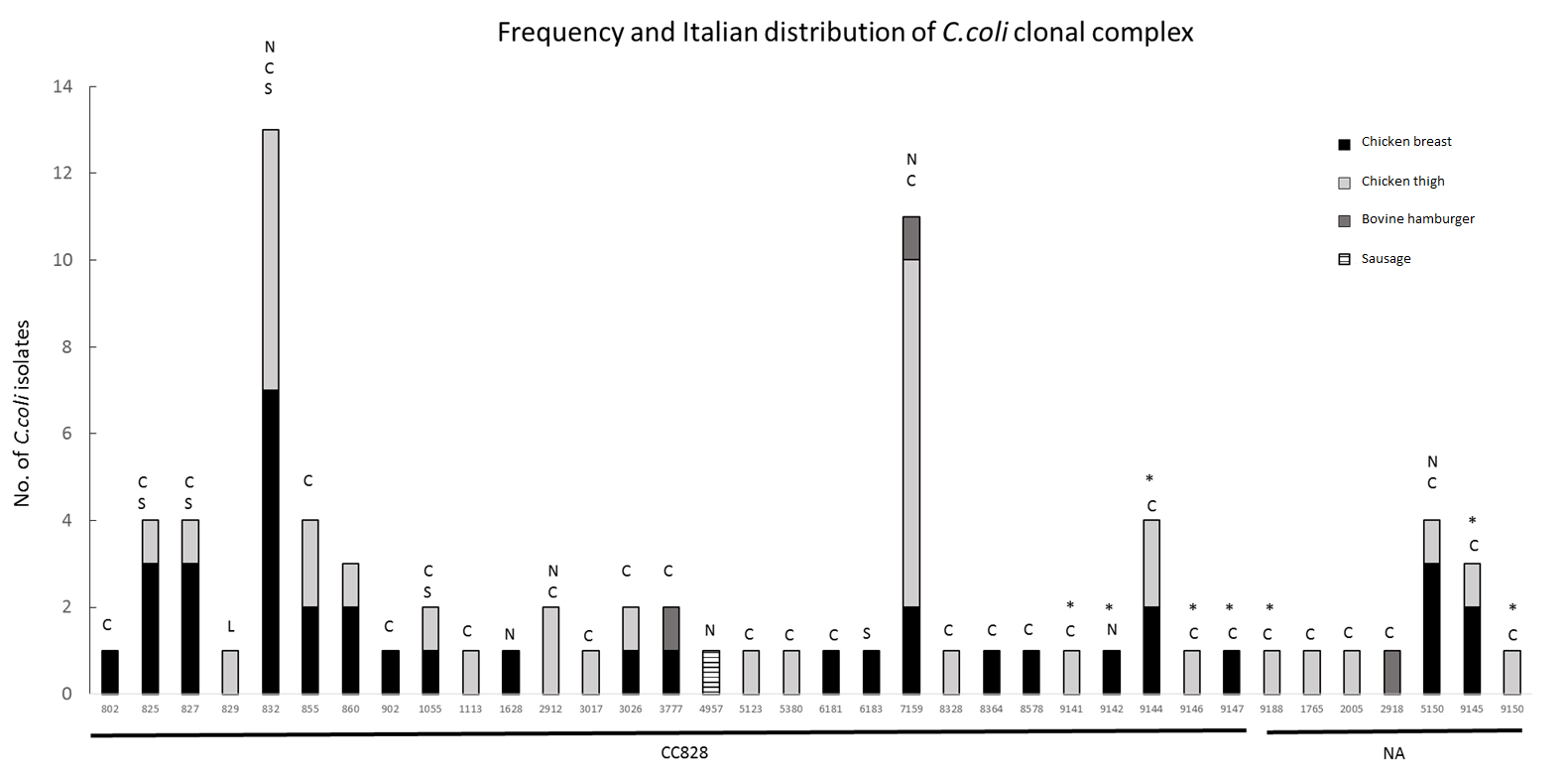

Supplement: S4 Fig — The capital letters above the histograms indicate the Italian regions of origin (N = Northern Italy, which includes Piemonte and Liguria regions; C = Central Italy, which includes Abruzzo, Lazio and Marche regions; S = Southern Italy, which includes Puglia and Basilicata regions). NA = CC not yet assigned. New STS found in this study are indicated in asterisk. (TIF) [file pone.0225957.s004.tif]
